# A systematic review of the effects of intimate partner violence on HIV-positive pregnant women in sub-Saharan Africa

**DOI:** 10.1186/s12889-022-12619-w

**Published:** 2022-02-03

**Authors:** Ashley Magero Yonga, Ligia Kiss, Kristine Husøy Onarheim

**Affiliations:** 1 Institute for Global Health, University College London, London, UK; 2 Gender Violence & Health Centre, Department of Global Health & Development, London School of Hygiene & Tropical Medicine, London, UK; 3 Department of Global Public Health and Primary Care, University of Bergen, Bergen, Norway

## Abstract

**Background:**

Intimate partner violence (IPV) affects more than one in three women in sub-Saharan Africa (SSA). It is associated with both pregnancy and HIV, adversely affecting women in this region. This is the first systematic examination of the effects of IPV on HIV-positive (HIV+) pregnant women in SSA.

**Methods:**

A systematic review of the literature on HIV+ pregnant women experiencing IPV in SSA was carried out. Searches were carried out in *PubMed, Web of Science and African Journals Online* databases. Articles published between January 2010 and June 2020, in English, were included. Data extraction included details on study locations, study design, study participants and the study outcome variables (depression, IPV, medication adherence, postpartum unsafe sex, and HIV disclosure).

**Results:**

Fourteen studies (ten cross-sectional studies, four cohort studies) were included. Results indicate a high prevalence of IPV amongst pregnant women with HIV in SSA (18.0 to 63.1%). The results suggest an association between HIV-positive status and consequences of IPV during pregnancy, particularly mental health effects, such as depression symptoms and suicidal ideation. HIV-related stigma has a key role within the relationship between HIV and IPV during pregnancy. One study described that the presence of IPV reduces adherence to Prevention of Mother-To-Child Transmission (PMTCT) medication. Three studies reported no association between HIV positive status or HIV status disclosure and IPV during pregnancy.

**Discussion/conclusions:**

The systematic review confirms interconnections between IPV and HIV seropositivity amongst pregnant women in SSA. Importantly, stigma, social isolation and poor mental health hinder help-seeking, disclosure, and treatment adherence among HIV+ pregnant women exposed to IPV in SSA. As a result, the potential of community interventions to tackle issues associated with IPV in HIV-positive pregnant women in this area should be explored in research, policy, and practice.

**Supplementary Information:**

The online version contains supplementary material available at 10.1186/s12889-022-12619-w.

## Background

### Introduction

Violence against women is one of the most pervasive manifestation of gender inequalities and violation of human rights [1, 2]. The most common form is intimate partner violence (IPV), with 1 in 3 women, globally, experiencing violence by a partner or ex-partner in their lifetime [2, 3]. IPV results in devastating consequences for women and their children and these effects have been well-documented [4–7], as have the associations between IPV exposure and positive HIV status [8–10].

In the WHO Africa region, the estimated prevalence of HIV of 36.6% (95% CI = 32.7-40.5%) is higher than the rate in high-income countries of 23.2% (95% CI = 20.2-26.2%) [6]. The high burden and detrimental effects of HIV in sub-Saharan Africa (SSA) are well-known [11–18], accounting for 71% of the global burden of disease of HIV [19]. However, a comprehensive understanding of the literature on IPV, HIV and pregnancy is missing. This paper aims to systematically review the literature on HIV+ pregnant women experiencing IPV in SSA. The review focuses on SSA due to the high prevalence of IPV, high prevalence of HIV seropositivity, shared risk factors for IPV and HIV and the substantial lack of literature on IPV in this region.

### Effects of IPV on health

Physical health consequences of IPV, regardless of pregnancy, include physical injuries, such as, bruises, broken ribs and head injuries [3, 20]. In addition, IPV is associated with long-term chronic health problems, including chronic pain and gastrointestinal disorders [3, 20], due to continued exposure to violence and stress. These health conditions persist even after exposure to violence has ended [20]. Mental health effects of IPV are also extensive and include depression, anxiety disorders, post-traumatic stress disorder (PTSD), sleep disturbances and suicide attempts [2, 5].

**Fig. 1:**
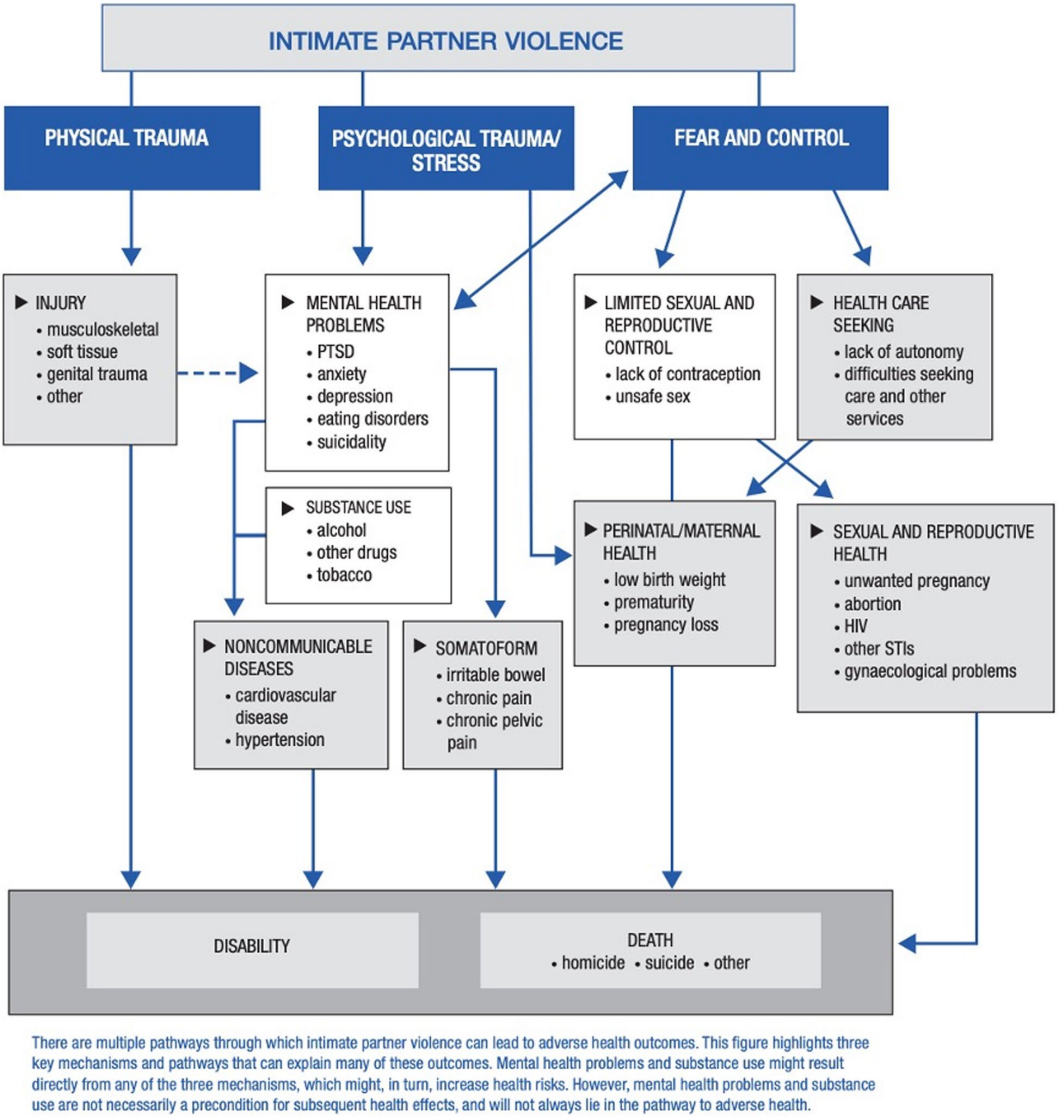
Pathways and health effects of IPV (Figure with permission from WHO [22])

IPV restricts health seeking behaviour as well as sexual and reproductive control for women. This puts them at increased risk for other health problems such as sexually transmitted infections (STIs), unwanted pregnancies, obstetric complications and long-term chronic diseases as a result of stress [5–7]. IPV during pregnancy can have direct repercussions on neonates, with high rates of low birth weight and preterm birth associated with IPV, potentially due to the physiological responses to the stress induced by violence exposure [6, 8, 9]. Furthermore, IPV has been reported to reduce women’s ability to care for their children [9], with particular impacts on health-seeking behaviours for their children [1, 21] and breastfeeding [5, 11]. Figure 1 depicts the complexity of IPV’s interaction with the physical, mental, sexual, and reproductive health of women.

While it is essential not to perpetuate harmful gender stereotypes by concentrating on women in relation to their reproductive potential [23, 24], IPV during pregnancy requires particular attention for four reasons. Firstly, pregnancy is a vulnerable time for women, making them more susceptible to harm [25]. Additionally, it affects two or more lives, with detrimental effects on mothers and neonates. Third, there is a wide variation of prevalence estimates for IPV during pregnancy; Devries et al. [26] discuss estimates of 3 to 30% from previous global prevalence studies. Finally, pregnancy can result in increased contact with healthcare services which allows for increased opportunities to screen for, detect and support women exposed to or at risk of IPV [27].

### HIV and IPV in sub-Saharan Africa

WHO and UNICEF data report that vertical transmission of HIV from mother to child accounts for approximately 35% of HIV infected infants worldwide [28]. An estimated 9 out of 10 of the over 2.8 million children and adolescents who are living with HIV in 2020 were living in SSA [29, 30]. Studies focusing on how this burden is related to IPV during pregnancy are limited [5]. The WHO recognises how violence against women and girls can contribute to their increased vulnerability to HIV, especially in this region [31]. They implicate the following links between HIV and IPV: direct transmission through sexual violence, increased HIV risk-taking behaviours by women because of previous or current exposure to violence, fear of discussing condom use and fear of violence if tested HIV+, preventing women from being tested or disclosing their status.

**Fig. 2:**
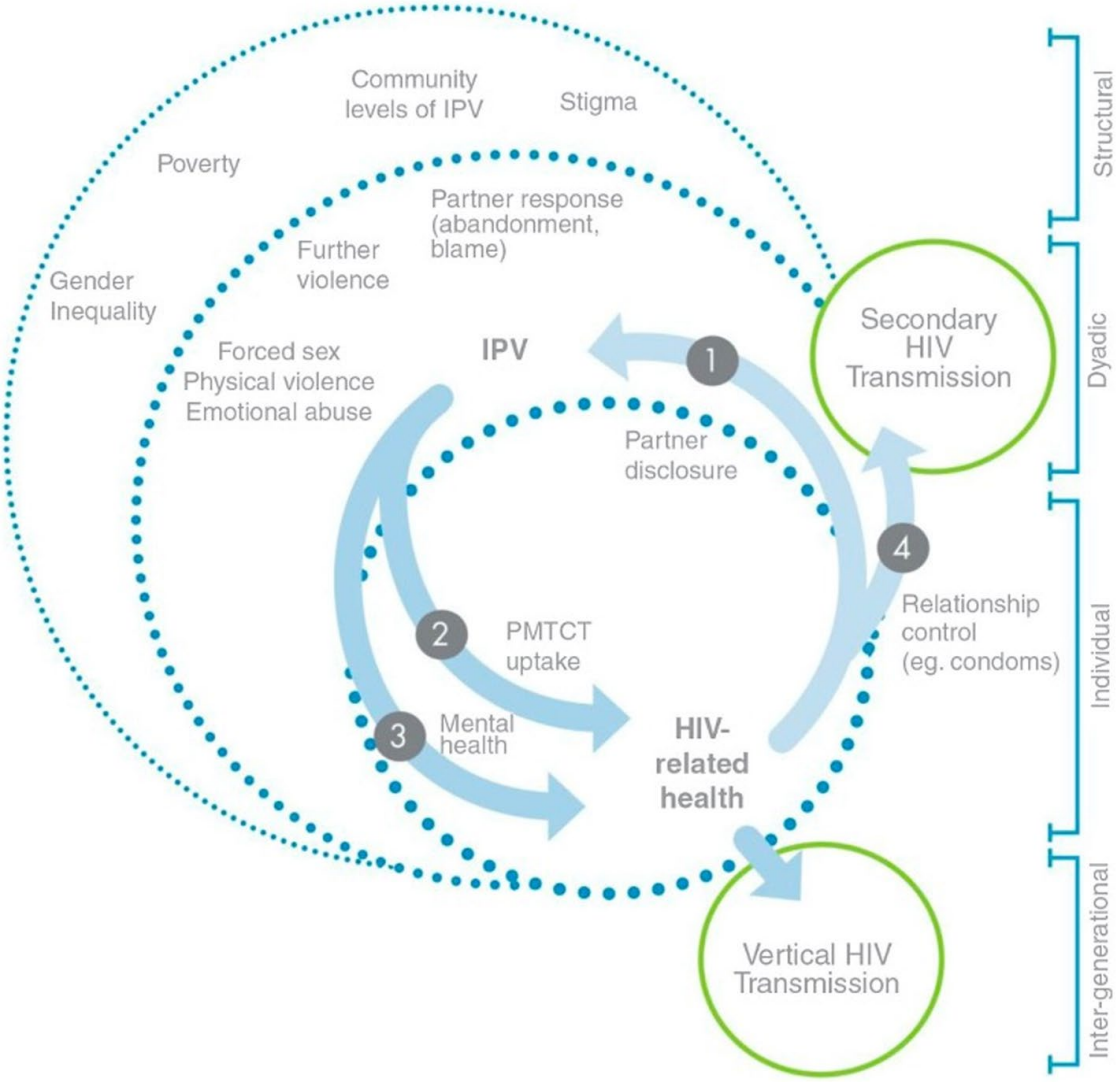
Figure available on Open Access from Hatcher and colleagues: IPV and HIV-related health issues among pregnant women [40]

Importantly, both HIV and IPV are associated with an overwhelming level of stigma [32–37], defined as having *‘a socially devalued identity’* which can be seen as a ‘*mark of failure or shame’* ( [34]: *p 2*). This stigma can be externally or internally imposed, that is, either from others or from oneself respectively [38, 39]. It creates barriers for reporting and healthcare seeking and adds to the physical and mental health burden of HIV and IPV individually [32, 34–37]. Figure 2 [40] demonstrates four ways that IPV and HIV seropositivity interact amongst pregnant women: partner disclosure, Prevention of Mother-To-Child Transmission (PMTCT) uptake, mental health and relationship control. These effects of IPV and HIV, and associated stigma, are likely compounded in SSA, where a large proportion of women live in poverty; 27 of the 28 poorest countries in the world are in this region [41].

The aim of this review is to examine this double burden of IPV and HIV through studying the existing literature on IPV effects on HIV+ pregnant women in SSA. This is to shed light on the specific challenges this vulnerable group faces.

## Methods

This systematic review followed the Preferred Reporting Items for Systematic Reviews and Meta-Analyses (PRISMA) guidelines (Additional files 1 and 3), informed by previous systematic reviews in this area [42–46].

### Search methods

Systematic searches were performed in three electronic databases: PubMed, Web of Science and African Journals Online. Separate search strategies were developed for each database including key words and MeSH terms [46]. Search terms included *intimate partner violence, domestic abuse, domestic violence, pregnancy, primigravida, HIV and human immunodeficiency virus* (Additional file 2). Articles included were published from January 2010 to June 2020. This period was chosen with interest in up-to-date studies reflecting the time window where access to antiretroviral therapy (ART) in low- and middle-income countries was available. The number of people on ART increased from 2 million to 17.2 million people between 2005 and 2010 [47].

### Selection process

All records captured by the search terms were exported to a Mendeley library. Duplicates were eliminated. Titles and abstracts were screened by the primary author. Those that did not meet the eligibility criteria were excluded. The remaining articles were examined through full-text review, with reasons for exclusion noted down, until a final set of studies to be included was established.

### Eligibility criteria

#### Inclusion criteria

The eligibility criteria were studies published between January 2010 to June 2020. Articles based on original quantitative research results, undertaken in an SSA country using any of the following study designs: cross sectional, cohort, case control, and randomised controlled trial. Only articles published in English in peer-reviewed journals were included. Studies had to include pregnant women (or mothers attending postnatal care reporting IPV during pregnancy). Articles had to focus on IPV only (physical, sexual, and emotional) and/or risk factors. IPV was defined as violence occurring in relation to past and current spouses, boyfriends, fiancés, whether they were married, cohabitating or dating [43]. Articles were included when reporting HIV status of either women or both men and women.

#### Exclusion criteria

Exclusion criteria were: papers focusing on domestic violence/abuse that was familial or parental; papers focusing on IPV during pregnancy in non-SSA countries; reporting on effects or experiences of others than women (e.g. focusing on neonates, infants, children or men only); grey literature, non-academic papers (commentaries, editorials, etc.), books, book chapters; other systematic reviews (in order to focus on primary data and avoid double reporting); qualitative studies; studies focusing on treatment/management of IPV only; studies where strength of associations (*p* values) were not reported; studies where the methods used are not adequately described, for instance questionnaire contents not reported, or the questionnaires were not validated.

### Data extraction

Following full-text analysis, data were extracted with the use of a data extraction form adapted from previous studies [43, 45]. Items included study locations, study design, study participants and outcomes. If two articles used the same data set but reported on different outcome variables, both articles were included if they met the eligibility criteria.

### Quality assessment and risk of bias

Selected studies were assessed for quality and risk of bias by the primary author in discussion with the co-authors. Quality assessment was informed by the criteria used by Alhabib et al. [48], which were adapted for the purposes of this paper:

- Target population explicitly stated
- Use of probability sampling methods e.g., simple random sampling, stratified sampling, clustered sampling
- Large enough sample size (≥ 300 participants)
- Reported and adequate response rate (≥80%)
- Reports the use of trained interviewers
- Reports confidence intervals or standard errors
- Reported attempt to reduce selection and/or measurement bias
- Adjusted for confounding variables.

Articles were scored for quality, where each criterion was worth 1 point. Hence each included article was given a score out of 8. A score equal to or less than 4 was classified as “poor”, 5-6 as “good” and 7-8 as “very good”.

## Results

### Study characteristics

**Fig. 3:**
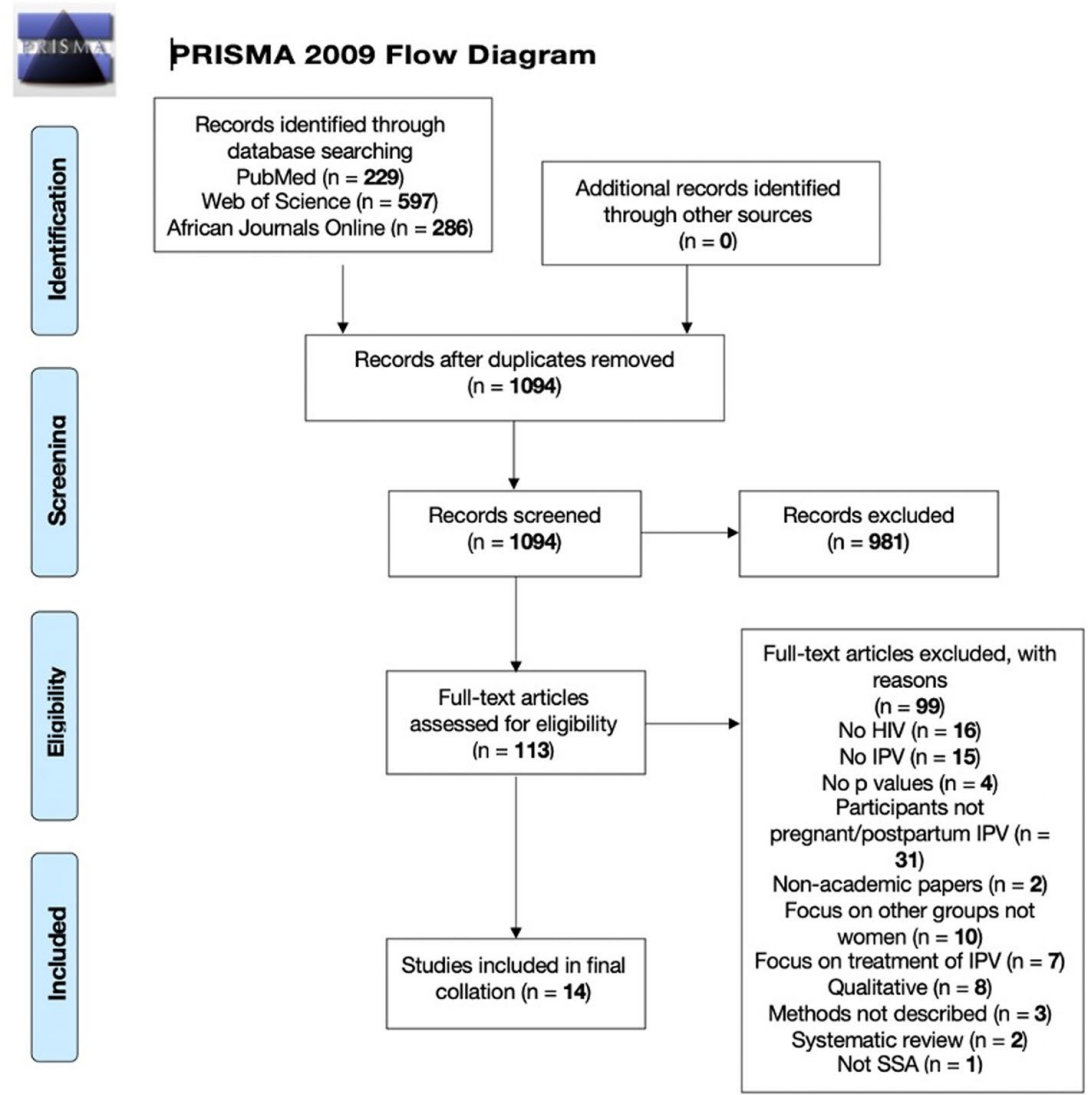
PRISMA flow diagram of the data extraction process [42]

The search strategy captured 1112 records. Eighteen duplicates were removed, and 1094 abstracts were screened, eliminating 981 for not meeting the eligibility criteria, therefore resulting in 113 for full-text review. The review includes 14 studies (Fig. 3**,** Table 1). The most common reason for exclusion was study participants not being pregnant or solely focusing on the postpartum period.

**Table 1:** Characteristics of included studies

No.	Author, year	Study location	Study design	Study participants	Main Outcomes	Alhabib Score
1	Bernstein et al., 2016 [49]	Primary care antenatal clinic Cape Town, **South Africa**	Cross-sectional	623 HIV-infected pregnant women aged between 18 and 44 years, from March to June 2013	IPV, depression, substance abuse and psychological distress	5
2	Hampanda, 2016 [50]	Large public health clinic in Lusaka, **Zambia**	Cross-sectional	320 HIV+ postpartum women from April to August 2014	Medication adherence during and after pregnancy	6
3	Manongi et al., 2017 [51]	Kilimanjaro Region, Northern **Tanzania**	Cross-sectional	1116 pregnant women attending antenatal care in Tanzania from March 1st, 2014 to May 30th 2015	Signs of depression during pregnancy	5
4	Matseke et al., 2016 [52]	Primary health care facilities in rural Mpumalanga, **South Africa**	Cross-sectional	673 HIV+ pregnant women	Physical and psychological IPV	7
5	McNaughton Reyes et al., 2020 [53]	Health clinic in KwaZulu-Natal, **South Africa**	Longitudinal cohort	T1 during pregnancy – 1480 women seeking antenatal care from the health clinic T2 (14 weeks postpartum) – 1154 women (78%) T3 (9 months postpartum) – 1104 (75%)	Postpartum emotional distress	4
6	Nyamukoho et al., 2019 [54]	Antenatal clinics in the Chitungwiza City Council outside of Harare, **Zimbabwe**	Cross-sectional	194 pregnant HIV+ women at Chitungwiza city council ANC clinics between 14 June 2016 to 14 September 2016	Prevalence of antenatal depression	5
7	Peltzer et al., 2018 [55]	12 Community health centres in Gert Sibande and Nkangala districts in Mpumalanga province, **South Africa**	Longitudinal cohort	681 women living with HIV were enrolled during pregnancy (8-24 weeks) and completed assessments at baseline; 32 weeks prenatally – 324 (47.6%) 6 months postnatally – 345 (50.6%) 12 months postnatally – 403 (59.2%)	Prevalence of prenatal and postpartum depression	6
8	Peltzer et al., 2020 [56]	12 community health centres in rural **South Africa**	Longitudinal cohort	1370 HIV-infected women enrolled at 8-24 weeks pregnant and followed postpartum at 6 weeks, 6 and 12 months Retention rate = 69.8% Recruited between April 10, 2014 and January 30, 2017	Depressive symptoms	7
9	Reyes et al., 2019 [57]	A healthcare clinic in KwaZulu Natal, **South Africa**	Longitudinal cohort	At baseline, 561 women diagnosed as HIV+ during pregnancy At 14 weeks postpartum – 421 retained (75%)	Postpartum unsafe sex	5
10	Rodriguez et al., 2017 [58]	Mpumalanga, **South Africa**	Cross-sectional	673 pregnant women living with HIV recruited from April 2014 to April 2015	Suicidal ideation	7
11	Shamu et al., 2013 [59]	Six low-income urban clinics in Harare, **Zimbabwe**	Cross-sectional	2042 women attending postnatal clinics between May and September 2011	IPV during pregnancy and risk factors	6
12	Wong et al., 2017 [60]	Guglethu Midwife Obstetric Unit in Cape Town, **South Africa**	Cross-sectional	625 HIV-infected pregnant women initiating antiretroviral therapy (ART)	Depression, alcohol use and stigma	7
13	Peltzer, Rodriguez and Jones 2016 [61]	12 community health centres in Mpumalanga province, **South Africa**	Cross-sectional	663 HIV+ prenatal women who were 20-24 weeks pregnant, recruited from a clinic RCT	Prenatal depression	7
14	Ramlagan et al., 2018 [62]	12 community health centers in Mpumalanga province, **South Africa**	Cross-sectional	673 HIV+ women, less than 6 months pregnant recruited from a clinic RCT – cross sectional data gathered from April 2014 to March 2015	Disclosure of HIV status	7

The earliest publication year was 2013, and the most common was 2016 (*n* = 4, 28.6%). Many of the studies were undertaken in South Africa (*n* = 10, 71.4%), followed by Zimbabwe (*n* = 2, 14.3%), Zambia (*n* = 1, 7.14%) and Tanzania (*n* = 1, 7.14%). Ten of the studies were cross-sectional (71.4%), while 4 were longitudinal cohort studies (28.6%). In relation to sample size, only one study had less than 300 participants. All studies collected data from clinical samples, except the research by Rodriguez et al. [58], which analysed population data from a province in South Africa. Questionnaires were used as the main data collection method in all studies; however, this was done in different ways: either face to face interviewing (*n* = 8 [49–51, 53, 54, 57, 59, 60]: or using Audio Computer-Assisted Self-Interview (ACASI; *n* = 6 [52, 55, 56, 58, 61, 62]. IPV was measured either through a WHO questionnaire (*n* = 6: studies [51, 53, 54, 56–58]) or the use of a Conflict Tactics Scale (*n* = 7: studies [52, 59–64]). A large proportion of the studies studied HIV+ women only (*n* = 11, 78.6%), despite the search strategy not differentiating between HIV+ and HIV-negative samples. Through quality assessment (Alhabib Score), one study was classified as “poor”, six as “good” and seven as “very good”.

Nine out of fourteen studies reported on mental health, three had IPV as one of the main outcomes, one reported on medication adherence, one on HIV status disclosure and one on postpartum unsafe sex. Eight of the papers included partner’s HIV status [50, 52, 55, 56, 58, 59, 61, 62], but none reported it as a main outcome. Table 1 presents a summary description of the included articles.

Overall, eight of the fourteen studies (57.1%) reported interactions between HIV+ status and IPV risk and/or effects during pregnancy. IPV prevalence reported in studies is summarised in Table 2.

**Table 2:** IPV prevalence of study populations, where reported

Study	Study location	Population	Prevalence IPV (%)
Bernstein et al. 2016 [49]	Primary care antenatal clinic Cape Town, **South Africa**	HIV+ pregnant women	21.0
Hampanda, 2016 [50]	Large public health clinic in Lusaka, **Zambia**	HIV+ postpartum women	61.0
Manongi et al., 2017 [51]	Kilimanjaro Region, Northern **Tanzania**	Pregnant women in antenatal care	38.8
Matseke et al., 2017 [52]	Primary healthcare facilities in rural Mpumalanga, **South Africa**	HIV+ pregnant women	56.3
McNaughton Reyes et al., 2020 [53]	Health clinic in KwaZulu-Natal, **South Africa**	Pregnant women in antenatal care	18.0
Nyamukoho et al., 2019 [54]	Antenatal clinics in the Chitungwiza City Council outside of Harare, **Zimbabwe**	Pregnant HIV+ women	22.7
Reyes et al., 2019 [57]	A healthcare clinic in KwaZulu Natal, **South Africa**	Women diagnosed as HIV+ during pregnancy	26.0
Shamu et al., 2013 [59]	Six low-income urban clinics in Harare, **Zimbabwe**	Women attending postnatal clinics	63.1
Wong et al., 2017 [60]	Guglethu Midwife Obstetric Unit in Cape Town, **South Africa**	HIV-infected pregnant women initiating antiretroviral therapy	21.0
Peltzer, Rodriguez and Jones, 2016 [61]	12 community health centres in Mpumalanga province, **South Africa**	HIV+ prenatal women	19.6
Ramlagan et al., 2018 [62]	12 community health centers in Mpumalanga province, **South Africa**	HIV+ women, less than 6 months pregnant	19.6

No association was found between IPV and disclosure of HIV status to a partner [OR 0.73 (95% CI 0.50-1.06; *p* = 0.112)] or someone else [OR 0.87 (95% CI 0.57-1.31; *p* = 0.499)] in one study [62]. Furthermore, one study [49] reported that there was no difference in IPV prevalence between women who had been diagnosed with HIV previously compared to those newly diagnosed during the current pregnancy. Another study [53] reported no interaction between HIV and IPV exposure during pregnancy.

IPV prevalence during pregnancy ranged from 18.0% [53] to 63.1% [59]. In the studies examining different forms of IPV, emotional/psychological violence was found to be the most prevalent [49–51]. Studies looking at mental health symptoms, reported strong associations between IPV and depression symptoms [49, 51, 52, 54, 55, 60, 61, 63], postpartum emotional distress [53] and suicidal ideation [58] among HIV+ pregnant women.

Peltzer, Rodriguez and Jones [61] highlight a key component within the relationship between IPV, HIV, mental health and unplanned pregnancy. Among women who reported IPV, the odds of depression were higher for HIV+ women with an unplanned pregnancy compared to those with a planned pregnancy (*p* < 0.001). Contrastingly, when IPV was not reported, this difference in odds of depression between these groups ceases (*p* > 0.05). Additionally, Wong et al. [60], found that amongst HIV+ women experiencing IPV, younger women (18-24 years old) had a higher mean depression score compared to older women (≥25 years old) – 9.8 vs 6.8 (*p* = 0.01). Both younger and older HIV+ pregnant women experiencing IPV reported more depressive symptoms compared to those not experiencing IPV [60].

In the nine studies whose main outcome was mental health, six reported some relationship between HIV+ status and depressive symptoms in pregnant women [51–53, 55, 56, 58]. Manongi et al. [51] reported HIV as an effect modifier for the association between emotional violence and signs of depression with the adjusted odds of experiencing emotional violence being 2.61 (95% CI 1.62-4.20), compared to those without HIV 0.09 (95% CI 0.00-3.16). HIV-related stigma was one of the key factors associated with depressive symptoms [56, 61] and suicidal ideation [58]. Matseke et al. [52] further reported an association between HIV-related stigma and any form of IPV.

Two of the studies found a significant association between IPV and HIV risk behaviour [50, 57]. Hampanda [50] demonstrated that the presence of IPV reduces adherence to PMTCT medication, reporting a dose-response relationship where for each additional violent event a woman experienced, she had 20% reduced odds of adherence to the medication during pregnancy. Severe psychological and physical IPV, coupled with male controlling behaviour, increased the odds of unsafe sex postpartum among HIV+ women in Reyes et al. [57]. The authors reported an adjusted odds ratio (aOR) of 7.19 (95% CI 1.60-32.33) among IPV survivors after controlling for sociodemographic variables and length of relationship. The aOR increased to 7.74 (95% CI 1.59-37.71) among women who experienced IPV after addition of theoretical confounders to the model, including childhood abuse and age at first sex. This study did not find a statistically significant association between moderate IPV and postpartum unsafe sex.

## Discussion

IPV exposure in HIV+ pregnant women poses a substantial health burden, especially in SSA. This is the first systematic review examining the effects of IPV on this group. There was a wide variety of reported IPV prevalence in the included studies of this review, with rates ranging from 19.6% [61, 62] to 56.3% [52]. This is likely due to the various challenges involved in determining IPV prevalence globally and more specifically in SSA [7, 20]. These are discussed in more detail in the limitations section.

The review’s findings demonstrate five key issues. Firstly, the relationship between IPV and the mental health outcomes of HIV+ pregnant women. Secondly, emotional/psychological violence was reported as the most prevalent form of IPV. Third, unplanned pregnancy was highlighted as a crucial factor in increased depressive symptoms in HIV+ pregnant women experiencing IPV. Fourth, stigma was shown to have a tremendous role to play within this relationship and finally, IPV increases HIV risk behaviours in this group, such as reducing condom use and reducing adherence to PMTCT medication. These findings align with the model from Hatcher et al. [40] that illustrates the interaction between IPV and HIV seropositivity.

Overall, the review describes a high prevalence, and thus a substantial burden, of IPV in HIV+ pregnant women in SSA, especially emotional/psychological abuse. It is crucial to note that it is usually more difficult to measure this form of IPV, since emotional/psychological abuse can be difficult to conceptualise [22, 27]. The nature of this type of abuse, however, could go some way in explaining its high prevalence of IPV in HIV+ pregnant women in SSA. Potentially, perpetrators and others may consider these behaviours more acceptable [27] and consequently, they may not view them as abuse. Further research in this area is necessary to explore emotional and psychological abuse in pregnant women in more depth.

Most studies focussed on the mental health of HIV+ pregnant women exposed to IPV, which is important as the search strategy did not target mental health explicitly. They report high levels of depressive symptoms, psychological distress, and suicidal ideation because of IPV exposure. Furthermore, HIV+ pregnant women exposed to IPV who are younger or who have unplanned pregnancies were at higher risk of increased depressive symptoms, compared to women who were not exposed to IPV. Ashaba et al. [64] corroborate this in their qualitative study. It demonstrated that increased verbal and emotional abuse was associated with unplanned pregnancy in women living with HIV, particularly when informing their partners about the unplanned pregnancy. This has implications for determining which groups to target within healthcare settings, and the role that sexual and reproductive health (SRH) services can play in IPV interventions. Additionally, Hatcher et al. [40] discuss how maternal mental health associated with IPV is often overlooked during maternal contact with health professionals, which could be an important point of entry for this group.

### The double burden of adversity

HIV-related stigma was highlighted as a crucial contributor to the IPV experiences of pregnant women in our study. This review revealed that stigma was associated with all forms of IPV [52] and depressive symptoms [56] within this population. The findings imply that it is not just the physical experience of HIV, IPV or both that causes difficulties, but also, the associated feelings of shame, which can be both internalised and externally imposed.

While external stigma is important, and worth highlighting, the effects of internalised stigma need emphasis as these can be particularly insidious. The negative impact of both IPV and HIV on a person’s self-identity needs to be specifically focused on, as it relates to their individual coping strategies. This can result in harmful consequences, for instance limiting help-seeking behaviour or treatment adherence [35–37]. This can be aggravated when these exposures co-occur, as shown by two of the studies included in this review [52, 56].

Labels such as ‘victim’ or ‘HIV-infected’ can affect how a person sees themselves and how society perceives them [33, 38, 39]. Even if IPV or HIV are not disclosed, internalised stigma can still impact a person’s ability to seek help, therefore jeopardising opportunities for diagnosis and management [38, 39], or worsening their mental health [38, 65, 66]. The presence of both HIV and IPV stigma in this review have been described to leave expectant and current mothers feeling isolated [67, 68], creating a barrier to both HIV-related and pregnancy-related care [68]. A crucial step in IPV management, after these services have been made available, is recognising the need for them. Unfortunately, internalised stigma can hamper this tremendously. Moreover, it is a difficult aspect of this relationship to target as it requires changing both social norms and personal beliefs.

As discussed, HIV- and IPV-related stigma can influence medication adherence. Others have demonstrated a complex relationship between IPV, mental health and HIV medication adherence [40, 67]. Hatcher et al. [67] highlight that poor adherence can be used as a means of self-harm, as women experiencing both of these conditions during pregnancy may feel overwhelmed and therefore have a desire to end their lives. Additionally, ever present thoughts and experiences of violence can result in forgetfulness – either forgetting to take their medication or forgetting to pick them up. This effect can be aggravated by the memory impairment and concentration problems characteristic of PTSD and depression. Both these mental health conditions are strongly associated with IPV and trauma [1, 2, 69], despite PTSD not being one of the reported outcomes in the studies included in this review. For some women, however, motherhood can provide resilience, allowing them to focus on their child’s wellbeing [67]. This may allow women to continue taking their medication, however, it does not necessarily reduce their exposure to IPV nor its devastating effects.

### IPV, HIV and fear

This review provided a better understanding of the relationship between HIV and IPV with fear acting as a potential mediating factor. The findings suggest that interactions between partner disclosure, PMTCT uptake, and relationship control are key in understanding the association between HIV and IPV during pregnancy [50, 57]. This is corroborated by previous qualitative research [40]. IPV and fear of IPV can result in women not feeling confident enough to request condom use [57], placing them at increased risk of HIV and other STIs [40, 68, 70]. Furthermore, Hampanda [50] demonstrates how IPV can reduce PMTCT medication adherence. A systematic review by Hatcher et al. [71], supports this as they report that the presence of IPV reduces adherence to antiretroviral therapy for HIV+ women.

There are various potential reasons for reduced medication adherence. Firstly, IPV can contribute to reduced adherence through direct partner control over access to HIV treatment [64, 67]. Second, fear of HIV disclosure can result in women avoiding taking their medication to prevent their partners finding out. Many studies have reported that this fear stems from a worry that their partners will consider them unfaithful, resulting in increased emotional or physical violence [40, 64, 70, 72–74]. While a study in this review [62] reported no association between IPV during pregnancy and HIV status disclosure, other studies have emphasised the importance of this relationship [40, 64, 72, 75, 76]. The potential impact of internalised stigma should also be highlighted here. This is because it can result in low self-esteem that means women do not feel capable of asserting safe sex practices or medication adherence. All in all, this review has demonstrated the variety of ways IPV complicates the experiences of HIV+ pregnant women.

### Strengths and limitations

This review has key strengths. First, it was a systematic review, using PRISMA guidelines, to study effects of IPV on HIV+ pregnant women in SSA. Second, the search was conducted in multiple databases resulting in a more comprehensive review. Additionally, the inclusion of African Journals Online ensured that studies from SSA were not unintentionally excluded due to publication bias. Lastly, the exclusion of studies not reporting *p* values or data collection methods and the use of standardized quality assessment [48] of the included studies ensured avoidance of low-quality studies, which strengthen the reliance on the study findings. However, the explicit focus on studies published in English, the last 10 years and, within the medical literature may have excluded relevant literature.

Despite the valuable information garnered from the 14 included studies, the studies have several limitations. Firstly, all studies relied heavily on self-reporting for their key outcomes which is liable to social desirability bias, recall bias and under-reporting, especially considering the stigma associated with both conditions. The studies that used ACASI went some way in overcoming this as its use can contribute to reduced social desirability bias, and the audio function makes it more appropriate in low-literacy populations, but only if used correctly [77]. Secondly, depression symptoms were measured using the Edinburgh Postnatal Depression Scale, without diagnostic review. Therefore, it does not provide a definitive diagnosis of depression as defined by either the Diagnostic and Statistical Manual of Mental Disorders or the International Classification of Disease-10. This may have led to some discrepancies between studies.

As mentioned previously, prevalence of IPV varied greatly amongst the included studies. This is likely to be because what constitutes as abuse is not standardised between and within countries. In addition, it is heavily reliant on self-reporting [7] and women recognising what they experience as IPV. Secondly, the methodology of data collection in studies differ, including questionnaires or face to face interviews. These have their own inherent biases and can also result in different responses [19]. Furthermore, psychological/emotional forms of abuse and controlling behaviours may not be recognised as IPV, which may contribute to underreporting [7, 20]. Challenges in studying IPV and HIV are therefore likely to have impacted the studies included in this systematic review.

In addition to this, many of the studies used non-proportional sampling, meaning that some individuals had no chance of being selected. Subsequently, this inhibited the ability to estimate the effects of sample error resulting in a non-representative sample and potentially non-generalisable results. Importantly, most studies (*n* = 13) recruited clinical samples and only one study offered population estimates on IPV. As a result, prevalence estimates cannot be generalised. Moreover, only 3 studies included HIV-negative women in their sample, so it is difficult to draw conclusions based on this evidence. There is a need for further comparative studies to better understand this relationship.

The review identified gaps in the existing literature. While physical violence and psychological/emotional violence were often reported in the included studies, there was limited information on sexual violence and male controlling behaviours. Furthermore, the studies that reported on mental health outcomes only discussed depressive symptoms and suicidal ideation. However, anxiety, PTSD and substance abuse are crucial mental health conditions that are likely to affect this group [1, 2, 69], but this was not captured in this review. Two studies reported on substance abuse [49] and alcohol use [60], but they did not explore how IPV affects these outcomes in HIV+ pregnant women. There is therefore a need to broaden the scope of the mental health outcomes while researching this group.

Finally, there are the limitations associated with the cross-sectional study design used in a large proportion of the studies. This hinders interpretation of causal links between IPV and HIV among pregnant women. The few longitudinal studies identified in the review opted for short follow-up periods determined by pregnancy status (pre- and post-partum), independent of changes to HIV status or IPV exposure. Finally, the geographical distribution of studies was limited, with all studies conducted in English speaking countries in SSA, and most of the studies being conducted in South Africa (*n* = 10). Nevertheless, this review provides a starting point for enhanced research into the effects of IPV exposure on HIV+ pregnant women in SSA.

### Implications of findings

The results of this systematic review build on previous literature on IPV and HIV [43, 78–80]. This review offers new insights into the severe problem of IPV amongst HIV+ pregnant women in SSA, with devastating consequences on their mental and physical health. These findings have important implications for further research, policy, and practice.

The review has identified important research gaps. The presence of IPV during pregnancy complicates maternal mental health extensively amongst HIV+ women [49, 51, 52, 54]. Hence, further research is required into better diagnosis and management of mental health issues related to these two stigmatising conditions, especially looking at anxiety, PTSD, and substance abuse. Despite a search strategy aiming to identify sexual violence and controlling behaviours by intimate partners as well, little information was found, which could be both a cause and a consequence of stigma. It is also important to highlight that women who may not seek healthcare services will likely be underrepresented in this research, as most of the studies focused on healthcare settings. Subsequently, more efforts are needed to gather data on the needs of this specific group, and, how best to provide IPV services for them.

Only one included study reported on PMTCT adherence, however, this is noteworthy considering that one of the main ways that HIV spreads in SSA is vertical transmission from mother to child; Hampanda [81] approximates that it accounts for 15% of the total global incidence. Therefore, further qualitative and quantitative research on the effects of IPV during pregnancy on PMTCT adherence is vital to inform policymaking and advocacy. This also further corroborates the value of adding discussions on IPV into sexual reproductive health and rights discourse.

More research is also required comparing HIV-negative and HIV+ women’s experiences of IPV, perhaps with case-control studies, as many of the studies only examined HIV+ women. Furthermore, all longitudinal studies identified in the review included women enrolled during pregnancy with short term follow-up pre- and post-partum. Forthcoming studies should follow women through an extended period of their reproductive life and/or include comparison groups. This could contribute to understanding potential causal links between pregnancy, IPV and HIV status in the longer-term.

The findings provide guidance for policy and practice. The WHO guidelines on responding to IPV [82] suggest the use of antenatal care for increased IPV screening and intervention opportunities. This review highlights the importance of developing and implementing guidelines that recognize and target the concerns of HIV+ pregnant women exposed to IPV, particularly by Ministries of Health in SSA countries. This also includes increasing resources for training health workers to overcome the barriers of stigma related to these two conditions, as ensuring that healthcare workers do not retraumatise their patients is essential [83]. Due to the highly stigmatising nature of both HIV and IPV, including IPV management in already existing services and structures can help to capture women in this vulnerable group.

It could be argued that policies in the WHO AFRO region and at country level should consider IPV screening for pregnant women attending HIV clinics. The results suggest that exploration of outreach activities or targeted interventions, sensitised to stigma, should be considered. Our findings have illustrated how the isolation associated with HIV and IPV amongst pregnant women in SSA can affect their health-seeking behaviour. Hence, ensuring IPV screening and management is a part of antenatal, HIV and SRH services could be of tremendous value to these women.

## Conclusions

The findings of this review have illustrated that in SSA, HIV+ pregnant women are experiencing high levels of IPV. IPV exposure in HIV+ pregnant women has detrimental effects on maternal mental health especially, as there is a high prevalence of depression and suicidal ideation in this population. Furthermore, HIV and IPV cause a double burden of adversity, interacting through stigma. The review revealed an intricate relationship between HIV medication adherence, mental health and IPV. These findings have substantial repercussions for both mothers and their children, as vertical transmission still contributes to a large proportion of new HIV infections in SSA.

HIV+ pregnant women experiencing IPV are a highly vulnerable and highly marginalised group. There is a need for more research on screening, diagnosis and management as well as increased attention in policy making for this group. IPV is a deeply traumatising and isolating experience, with a large presence in SSA. Addressing IPV, HIV and their related stigma is long overdue to guarantee that every woman, pregnant or not, can feel safe in her own home.

## Supplementary Information

**Additional file 1.**

**Additional file 2.**

**Additional file 3.**

## Data Availability

All data generated or analysed during this study are included in this published article [and its supplementary information files].

## Abbreviations

IPV: Intimate Partner Violence
HIV: Human Immunodeficiency Virus
WHO: World Health Organization
SSA: Sub-Saharan Africa
PTSD: Post Traumatic Stress Disorder
STIs: Sexually Transmitted Infections
HIV +: HIV positive
PRISMA: Preferred Reporting Items for Systematic Reviews and Meta-Analyses
ART: Antiretroviral Therapy
ACASI: Audio Computer-Assisted Self-Interview
PMTCT: Prevention of Mother-To-Child Transmission
aOR: Adjusted Odds Ratio
SRH: Sexual and Reproductive Health
