# Supplementary material for: A systematic review of the effects of intimate partner violence on HIV-positive pregnant women in sub-Saharan Africa
Source: BMC Public Health. 2022 Feb 3;22:220. doi: 10.1186/s12889-022-12619-w (PMC8815228; doi:10.1186/s12889-022-12619-w)
Supplement: Supplementary file 2 — Additional file 2. [file 12889_2022_12619_MOESM2_ESM.pdf]

PubMed	Web of Science	African Journals Online
“intimate partner violence”[tiab] OR IPV [tiab] OR “intimate-partner violence”[tiab] OR “intimate partner abuse”[tiab] OR “domestic abuse”[tiab] OR “domestic violence”[tiab] OR “domestic assault”[tiab] OR “partner violence”[tiab] OR "Intimate Partner Violence"[**Mesh**]<br>**AND**<br>“pregna*” [tiab] OR “matern*” [tiab] OR “mother” [tiab] OR “primigravida” [tiab] OR "Pregnancy" [**Mesh**]<br>**AND**<br>“HIV” [tiab] OR “AIDS” [tiab] OR “human immunodeficiency virus” [tiab] OR “acquired immune deficiency syndrome” [tiab] OR "HIV"[**Mesh**]	intimate partner violence OR IPV OR intimate-partner violence OR intimate partner abuse OR domestic abuse OR domestic violence OR domestic assault OR partner violence<br>**AND**<br>pregna* OR matern* OR mother OR primigravida<br>**AND**<br>HIV OR AIDS OR human immunodeficiency virus OR acquired immune deficiency syndrome	“intimate partner violence” **AND** pregnancy **AND** HIV

Supplementary Material 2. Search strategy for each database (PubMed, Web of Science and African Journals Online) using key words and MESH terms.
