# Supplementary material for: A systematic review of the effects of intimate partner violence on HIV-positive pregnant women in sub-Saharan Africa
Source: BMC Public Health. 2022 Feb 3;22:220. doi: 10.1186/s12889-022-12619-w (PMC8815228; doi:10.1186/s12889-022-12619-w)
Supplement: Supplementary file 3 — Additional file 3. [file 12889_2022_12619_MOESM3_ESM.docx]

**Systematic Review Protocol**

| **Title of Review** | A systematic review of THE EFFECTS OF Intimate Partner Violence ON HIV-POSITIVE PREGNANT WOMEN IN SUB-SAHARAN AFRICA. |
| --- | --- |
| **Background to review** | |
| It is estimated that, globally, 1 in 3 women experience physical and/or sexual violence during their lifetime, (WHO, 2017) with the most common form being intimate partner violence (IPV) (Matseke, Peltzer and Habil, 2013; Undie *et al.*, 2016; Sigalla *et al.*, 2017; WHO, 2017). Shamu *et al.* (2011) report that pregnant women are at a higher risk of IPV, highlighting it as a serious health concern affecting millions of women and their children (Alhusen *et al.*, 2015; Hill *et al.*, 2016; Mahenge *et al.*, 2016; Sigalla *et al.*, 2017). However, IPV and its effects on maternal health, both physical and mental, is under-funded and under-explored in global health. Subsequently, it requires further examination and increased global attention.  Within the existing literature, systematic reviews and meta analyses have examined the effects of IPV on pregnancy and neonatal outcomes (Hill *et al.*, 2016; Halim *et al.*, 2018). To my knowledge, there is no review on IPV during pregnancy and HIV. Hence, this dissertation will focus on examining how HIV status, of both the pregnant woman and/or her partner, affects women’s experiences of IPV during pregnancy. Additionally, Sub-Saharan Africa is the chosen focus due to its high prevalence of IPV, (Undie *et al.*, 2016; Sigalla *et al.*, 2017), a highlighted lack of research into IPV during pregnancy (Shamu *et al.*, 2011), and a high prevalence of HIV (Avert, 2018). Russell, Eaton and Petersen-Williams (2013) highlight the importance of examining the ways in which health issues intersect as an awareness of shared risk factors can assist in better comprehension and tackling of these issues. Consequently, it is vital to look closer at the relationship between these three critical issues: pregnancy, IPV and HIV. | |
| **Aim of review** | To collate the literature on the effects of IPV on HIV-positive pregnant women in sub-Saharan Africa. |
| **Specific objectives** | 1. Types of IPV prevalent amongst HIV+ pregnant women 2. How a new HIV+ diagnosis and/or disclosure impact IPV during pregnancy 3. The effects of IPV during pregnancy on maternal health in HIV+ women. |
| **Inclusion criteria** | Studies reporting IPV during pregnancy and HIV status of either men or women or both in sub-Saharan African countries. Studies published between January 2010 and June 2020 and in English. Published peer reviewed academic research papers, including both qualitative and quantitative studies. |
| **Exclusion criteria** | Papers that are: focusing on IPV during pregnancy in non-African countries; reporting on effects or experiences of others than women (e.g. focusing on neonates, infants or children **only**); grey literature, non-academic papers (commentaries, editorials, etc.), books, book chapters |
| **Search methods** | |
| **Electronic databases** | Web of Science  PubMed/Medline  African Journals Online |
| **Other methods used to identify relevant research** | N/A |
| **Search terms** | |
| **PubMed** | “intimate partner violence”[tiab] OR IPV [tiab] OR “intimate-partner violence”[tiab] OR “intimate partner abuse”[tiab] OR “domestic abuse”[tiab] OR “domestic violence”[tiab] OR “domestic assault”[tiab] OR “partner violence”[tiab] OR "Intimate Partner Violence"[Mesh]  **AND**  “pregna*” [tiab] OR “matern*” [tiab] OR “mother” [tiab] OR “primigravida” [tiab] OR "Pregnancy" [Mesh]  **AND**  “HIV” [tiab] OR “AIDS” [tiab] OR “human immunodeficiency virus” [tiab] OR “acquired immune deficiency syndrome” [tiab] OR "HIV"[Mesh] |
| **Web of Science** | intimate partner violence OR IPV OR intimate-partner violence OR intimate partner abuse OR domestic abuse OR domestic violence OR domestic assault OR partner violence  **AND**  pregna* OR matern* OR mother OR primigravida  **AND**  HIV OR AIDS OR human immunodeficiency virus OR acquired immune deficiency syndrome |
| **African Journals Online** | “intimate partner violence” AND pregnancy AND HIV |
| **Methods of review** | |
| **Details of methods** | Using MeSH and key terms generated from background/scoping literature; handsearching references; initial screening using titles and abstracts; detailed full-paper screening using critical appraisal tools; data extraction of included studies; narrative synthesis |
| **Quality assessment** | Critical Appraisal Skills Programme (CASP) Checklist for relevant type of study in each research paper – <https://casp-uk.net/casp-tools-checklists/>  BMJ Open Appraisal tool for Cross-Sectional Studies |
| **Data extraction** | Data extraction form based on existing literature – Excel spreadsheet  Mendeley Desktop App used to keep track of references |
| **Narrative synthesis –** details of what and how synthesis will be done | 1. Describing the theory and empirical findings of the interactions between IPV, pregnancy and HIV status 2. Developing a preliminary synthesis of findings of included studies 3. Describing the interactions within and between studies 4. Quality assessment of the synthesis |
| **Presentation of results** | |
| **Additional materials** | PRISMA Flow chart  Protocol  Data extraction form and tables |
